# Study of the Absorption Energy of Auxetic Structures with Non-Newtonian Fluids

**DOI:** 10.3390/ma18061263

**Published:** 2025-03-13

**Authors:** Daniel Barros, Carlos Mota, João Bessa, Fernando Cunha, Nelson Oliveira, Raul Fangueiro

**Affiliations:** 1 Fibrenamics, University of Minho, 4800-058 Guimarães, Portugal; carlosmota@fibrenamics.com (C.M.); joaobessa@fibrenamics.com (J.B.); fernandocunha@fibrenamics.com (F.C.); rfangueiro@fibrenamics.com (R.F.); 2 Spacengineer (SE), 3030-199 Coimbra, Portugal; nelson.oliveira@spacengineer.com; 3 Department of Textile Engineering, University of Minho, 4800-058 Guimarães, Portugal

**Keywords:** impact energy, auxetic, shear thickness fluids, absorption

## Abstract

Ballistic impact protection has been enhanced through the use of advanced materials, such as shear thickening fluids (STFs) and auxetic structures. These materials provide high energy absorption, flexibility, and comfort, offering promising solutions for the development of lightweight and effective personal protective equipment. The combination of STFs and auxetic structures has been shown to optimize impact resistance while maintaining mobility. To validate this, a composite made of an auxetic structure impregnated with a polyurethane and STF mixture was evaluated for energy absorption. The auxetic structure, fabricated using high-tenacity polyester, demonstrated superior energy absorption compared to standard foams. The impregnation of the auxetic structure with 200 and 400 wt% Biresin and STF mixtures significantly enhanced its impact energy absorption capacity up to 76% compared to the auxetic reference. With the addition of the STF at a 25:75 ratio into the biresin matrix, improvements were also verified in the absorption, up to 7%, due to the non-Newtonian behavior of the STF, demonstrating the potential of these composites for low-impact applications.

## 1. Introduction

Protection against ballistic impacts presents a significant challenge in the development of personal safety equipment, such as ballistic vests, helmets, and joint protectors. The increasing sophistication of threats, combined with the need for lightweight, flexible, and comfortable solutions, has driven research into advanced materials that combine high energy absorption capacity with seamless integration into existing systems [1,2,3]. In this context, materials such as non-Newtonian fluids, auxetic structures, and hybrid composites have emerged as promising alternatives, offering unique properties that can be leveraged to optimize protection against both low- and high-energy impacts [4,5,6].

Non-Newtonian fluids, particularly shear thickening fluids (STFs), have garnered considerable attention due to their unique rheological behavior. Under high shear rates, these materials exhibit a significant increase in viscosity, resulting in momentary resistance to deformation [7,8,9]. This property makes STFs ideal for applications requiring adaptive protection, such as mitigating ballistic impacts or fragments from explosions [10,11]. When impregnated into fibrous substrates, STFs also contribute to enhancing structural stiffness and energy absorption while maintaining a degree of flexibility and comfort.

Simultaneously, auxetic structures exhibit unique geometric and mechanical properties, characterized by a negative Poisson’s ratio. This means that, under tension, these structures expand laterally, in contrast to conventional materials that contract [12,13]. This response increases local density and enhances energy dispersion, making these structures highly effective in applications requiring impact resistance [14,15]. Combining three-dimensional auxetic structures with STFs, in turn, offers significant potential for creating composite materials that maximize energy absorption while preserving the flexibility necessary for mobility applications [16,17].

Previous studies on STF-impregnated fibrous structures have typically employed an approach where the fluid is applied to the substrate after its fabrication. While effective, this method presents limitations in terms of the homogeneity of fluid distribution and the ability to tailor it to different performance requirements [18]. Alternatively, direct mixing of the STF with the matrix during the material fabrication process can provide better control over fluid distribution and integration, enhancing the properties of the final material. This approach enables the exploration of various composite combinations, fine-tuning the balance between flexibility, stiffness, and energy absorption [19].

In this context, the present study aims to evaluate the energy absorption capacity of three-dimensional auxetic structures impregnated with different quantities of STFs. The primary focus is to develop solutions with high potential for low-energy impact absorption, aligning with the requirements of National Institute of Justice (NIJ) standards for personal protective equipment. Impact tests, including low-energy absorption tests and dynamic mechanical analysis (DMA), will be conducted to characterize the properties of the developed solutions across different frequencies of applied stress. Additionally, the chemical composition of the fabricated materials will be investigated, emphasizing the homogeneous integration of the STF into the structural matrix.

This study aims to advance the state of the art in impact protection materials, providing a solid foundation for the integration of these solutions into ballistic protection applications, ensuring enhanced performance and comfort for the end-user.

## 2. Materials and Methods

### 2.1. Materials

The composite samples were fabricated using a 3D auxetic structure made of polyester fibers with a diameter of 50 µm, a weight of 414 g/m^2^, and a thickness of 3.3 mm. These fibers were supplied by Fibrauto, Gaia, Portugal. The matrix used was a polyurethane combination, specifically Biresin U1419/U1458 from Sika^®^, acquired in Rebelco, Cascais, Portugal. Additionally, a shear thickening fluid (STF), produced and supplied by SpacEngineer, Coimbra, Portugal, was incorporated as an additive to enhance the material’s performance.

The shear thickening fluid (STF) used in this study was supplied by a partner organization. Due to confidentiality agreements, limited information about its detailed properties can be disclosed. Currently, the entity only allows us to share that the viscosity values of this STF, under testing conditions of a temperature sweep from 100 to 200 °C (temperature range specified by the client), in oscillatory mode, at a constant deformation of 1% and a frequency of 1 Hz, are as follows: at 100 °C, the viscosity is 30.58 Pa·s, and at 200 °C, it is 0.92 Pa·s. However, it is important to note that, unlike other solutions on the market, this STF is not a colloidal fluid composed of various solid particles (e.g., calcium carbonate, silica) and dispersing liquids (e.g., water, polyethylene glycol, ethylene glycol). Instead, the STF utilized in this study is based on a co-polymer molecular chain, incorporating several different monomers to achieve the desired rheological behavior.

### 2.2. Sample Preparation

The auxetic structures were impregnated with different matrix compositions, including pure polyurethane resin (PUR) and pure shear thickening fluid (STF) at mass percentages of 200% and 400%. Additionally, structures were impregnated with a mixture of PUR and STF in a mass ratio of 75:25, with both components applied at 200 wt% and 400 wt% relative to the mass of the auxetic structure. The percentages of 200% and 400% were chosen for impregnation due to the low-density nature of the structure being tested. As the resins and STF used as the matrix are denser, selecting impregnation percentages higher than 200% was the only way to ensure excellent and homogeneous impregnation.

For the PUR-STF mixture, the STF was first incorporated into both components of the resin (A and B). After the mechanical mixing of the two resin components for 15 min, the STF mixture was then combined with the resin components through further mechanical mixing for an additional 2 min. Each auxetic structure was then manually impregnated using a metal roller to ensure uniform distribution of the impregnation.

Following impregnation, each sample was subjected to heating in an oven at 80 °C for 2 h to facilitate resin curing, resulting in the formation of the auxetic composite structure.

**Figure 1:**
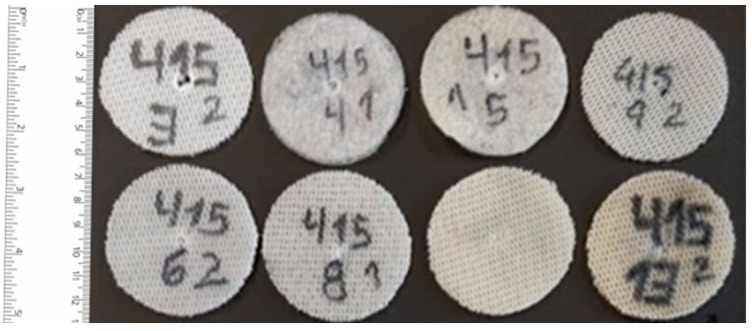
Schematic illustration of the sample production.

Table 1 displays all the samples produced, and Figure 1 shows the resulting cut samples.

## 3. Testing and Characterization

### 3.1. Fourier Transform Infrared Spectroscopy (FTIR) Procedure

The chemical composition of the resins and resin-STF mixtures used to impregnate the auxetic structures as matrix materials was analyzed using Fourier transform infrared spectroscopy (FTIR) coupled with the Attenuated Total Reflection (ATR) technique, utilizing a SHIMADZU—IRAffinity-1S instrument (Kyoto, Japan). Spectra were collected in transmittance mode with 45 scans across a wavenumber range of 4000–400 cm⁻^1^. This technique allows for the comparison of the chemical compositions of the selected resin and STF, as well as the spectrum of the resin-STF mixture.

### 3.2. Dynamic Mechanical Analysis Procedure

Dynamic Mechanical Analysis (DMA) is a widely used technique for characterizing the mechanical properties of materials under varying conditions, such as temperature, time, frequency, stress, and atmosphere. This method provides valuable insights, including the storage modulus, loss modulus, and Tan delta. The storage modulus reflects the material’s elasticity and its ability to store energy, while the loss modulus indicates its capacity to dissipate energy, typically in the form of heat. Tan delta measures the material’s damping properties, such as its ability to dampen vibrations or sound. To evaluate the properties of the shear thickening fluid (STF) and compare them with other materials, samples were analyzed at room temperature (~25 °C), focusing on the storage modulus at different compression frequencies of 1, 50, and 100 Hz.

### 3.3. Energy Absorption Procedure

To assess the energy absorption capacity of the different composites produced with various matrices, impact absorption tests were performed following the ISO 20344-5.17-2011 standard [20]. These tests were conducted at the Centro Tecnológico do Calçado de Portugal (CTCP) in S. João da Madeira, Portugal. Disks with a diameter of 60 mm and a thickness of 3 mm were cut from the produced samples. Each sample underwent puncture impact testing, with a holder placed beneath to measure the impact energy that was not absorbed by the sample. The puncture force applied in all tests was 98 kN. During the impact, each sample absorbed a portion of the energy, while the sensor in the sample holder recorded the force that remained unabsorbed.

## 4. Results and Discussion

### 4.1. Fourier Transform Infrared Spectroscopy (FTIR) Results

**Figure 2:**
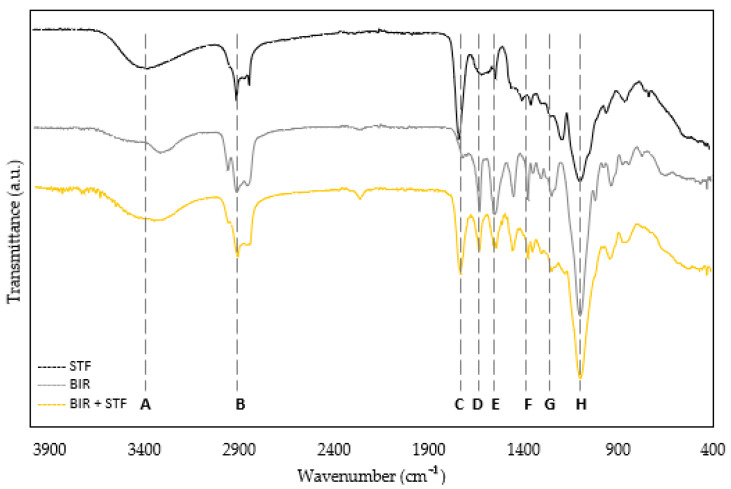
Fourier transform infrared spectra of matrices used.

FTIR analyses were conducted on the STF, Biresin resin, and their mixture after curing. The obtained FTIR spectra of the analyzed samples are depicted in Figure 2, and the identified significant bands are summarized in Table 2. As explained in the response to the reviewer’s comment, the FTIR analysis was performed to assess the compatibility between the STF and the resin, as the STF was added directly to the resin during the curing process. This approach differs from the typical method used in other studies, where the STF is initially added to the reinforcement and the matrix is introduced afterward.

From Figure 2 and Table 2, several significant bands can be identified across all tested matrices. Most of the peaks are common to all samples. The first peak appears at 3400 cm^−1^, typically attributed to the O-H stretching vibration. A second peak appears at 2920 cm^−1^, corresponding to the C-H asymmetric stretching vibration. Bands at 1711 cm^−1^ and 1626 cm^−1^ are associated with the C=O aldehyde and C=C alkene stretching vibrations, respectively. The band at 1528 cm^−1^ is attributed to an amide N-H bending vibration, and the 1385 cm^−1^ band corresponds to a C-H symmetric vibration. Finally, the bands at 1230 and 1093 cm^−1^ are typically linked to C-O aliphatic ether stretching vibrations.

The FTIR spectra obtained for the resin analyzed, when compared to the literature spectra, confirm that the material is polyurethane [21,22,23]. The spectra of the Biresin/STF mixture show a combination of the bands found in the individual spectra of each material. For instance, the mixture spectrum exhibits peaks B and C, which are attributed to the STF (as seen in its individual spectrum), and peaks D and E, which correspond to the Biresin (as seen in its individual spectrum). Upon examining the spectra of the mixture, we can identify characteristic bands from both STF and Biresin, as well as overlapping bands. This indicates compatibility between the two materials, which likely explains the successful curing of the Biresin resin after being mixed with the STF.

### 4.2. DMA Results

**Figure 3:**
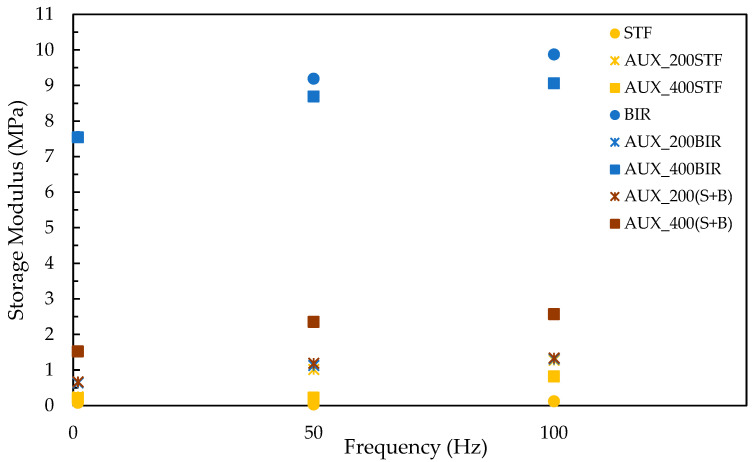
Storage modulus for different compressive solicitations.

The DMA test is essential for assessing a material’s response to impact, as it provides valuable insights into the material’s ability to absorb and dissipate impact energy efficiently. The material’s behavior across different frequencies during the DMA test is illustrated in Figure 3.

For the testing, specimens with a diameter of 10 mm and a thickness of 3 mm were used. During the definition of test parameters, it was observed that the materials are sensitive to the degree of deformation applied. As a result, a deformation amplitude of 25 microns was chosen to ensure accurate results. However, despite several attempts to optimize the test parameters, it was not possible to obtain results for the control samples (sponge and auxetic structure without matrix). This failure was likely due to the low modulus and high flexibility of these samples, which made it difficult to stabilize the initial stage of the test and obtain reliable readings.

The DMA results for all tested samples show a clear trend: for materials with good energy absorption capacity, the storage modulus increases with frequency. The shear thickening fluid (STF) samples exhibited the lowest modulus values. However, when the STF was incorporated into an auxetic structure, the modulus increased significantly, from 0.12 MPa to 0.82 MPa, a 683% increase. This enhancement in modulus highlights the synergistic effect of combining STF with auxetic structures, which improves the material’s mechanical properties.

Further improvements were observed when Biresin was added to the STF in a 75:25 ratio. The resulting auxetic/Biresin/STF composite demonstrated a modulus as high as 2.57 MPa, indicating a substantial increase in stiffness compared to the pure STF and auxetic combination. Among all the combinations, the highest modulus was found in the Biresin-impregnated auxetic structure with 400 wt% of Biresin, showcasing the significant contribution of Biresin to the composite’s mechanical performance

### 4.3. Energy Absorption Results

These tests were performed under low-impact conditions using a standard for clothing applications. The goal was to assess the energy absorption of the different samples and identify the best-performing ones. These selected samples will be further developed into prototypes and tested according to military clothing standards.

**Figure 4:**
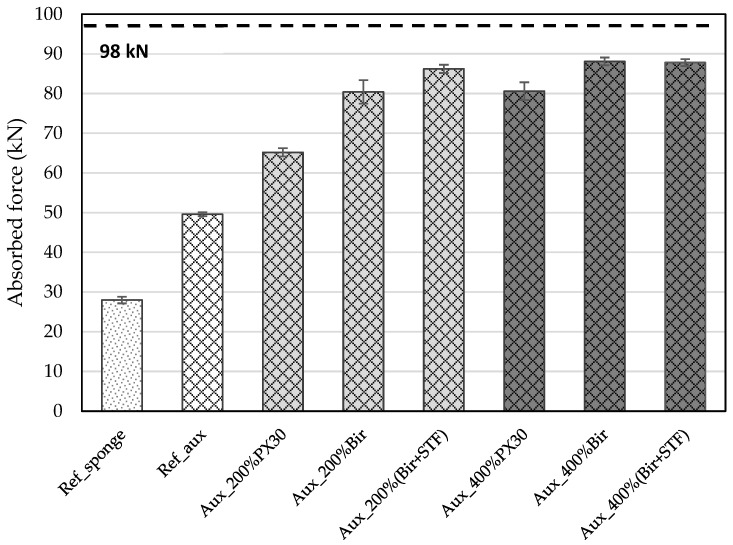
Force absorption measured values for each sample combination.

Each sample disk was subjected to an impact force of 98 kN, and the absorbed force for each was evaluated. The measured values for each tested combination are shown in Figure 4.

The results obtained show that the auxetic structure absorbs 50 kN of the 98 kN applied, while the reference sponge absorbs approximately 28 kN. Therefore, it can be concluded that the auxetic structure absorbs about 44% more force compared to the reference sponge.

The auxetic structures impregnated with the STF at 200 and 400 wt% absorbed approximately 42 and 48 kN, respectively, out of the 98 kN applied. This represents a decrease in absorbed impact of about 4% and 16% compared to the reference auxetic material. This reduction may be attributed to the large spaces within the auxetic structure, which could cause the STF particles to separate, leading to a loss of their non-Newtonian properties. As the STF content increases, more particles may interact, resulting in a more pronounced non-Newtonian behavior.

Comparing the combinations of Aux_200%Bir and Aux_200% (Bir + STF), it is evident that impregnation of the auxetic structure increases the force absorption. The first combination absorbed approximately 80 kN of the 98 kN applied, while the second absorbed around 85 kN. Thus, the Aux_200% (Bir + STF) exhibited a higher absorption force, approximately 7% more compared to the Aux_200%Bir. Furthermore, when compared to the Ref_aux, the Aux_200% (Bir + STF) demonstrated a 70% increase in absorption capacity.

For the auxetic structures impregnated with 400 wt% of Biresin and Biresin + STF, there was no significant difference in the absorption forces measured, with both combinations absorbing about 88 to 90% of the applied impact force. Compared to the Ref_aux, the Aux_400% (Bir + STF) showed a 76% increase in absorption capacity.

When comparing the Aux_200% (Bir + STF) sample to the Aux_400% (Bir + STF), the absorption capacity increased from 85 kN to 88 kN, representing a slight difference of about 3.5%. This could be due to the saturation of the auxetic structure’s “porosity”, reaching its maximum energy absorption capacity.

## 5. Conclusions

This study explored the development of auxetic composite structures through impregnation with polyurethane-based matrices and the addition of shear-thickening fluids (STFs), aiming to enhance energy absorption properties for low-impact energy applications. FTIR results demonstrated excellent chemical compatibility between the STF and polyurethane resin when mixed before the curing process, highlighting the feasibility of this method to achieve a homogeneous mixture and distribution of the STF within the matrix.

DMA results further showed that the addition of the STF and Biresin significantly enhanced the mechanical properties of the auxetic structures, while control samples were unsuitable for testing due to their inherent flexibility and low modulus. In impact absorption tests, the auxetic structure exhibited superior energy absorption compared to the reference sponge. Among the combinations tested, the auxetic structure impregnated with 400 wt% Biresin and the mixture of Biresin with the STF showed the best energy absorption properties. Additionally, the incorporation of the STF into the auxetic structure with 200 wt% of polyurethane increased its absorption capacity, indicating the positive effect of the STF on energy dissipation.

In conclusion, the impregnation of the auxetic structure with resin and STF mixtures, particularly at 200 and 400 wt%, significantly enhanced the impact energy absorption capacity. The addition of the STF, mixed with the resin at a 25:75 ratio, contributed to an increase in energy absorption, attributed to the non-Newtonian behavior of the STF. This work demonstrates the potential of auxetic structures combined with STF-impregnated polyurethane matrices for improved energy absorption in low-impact applications.

As future work, we will focus on developing new compositions using the auxetic structure, both with and without STF addition, based on the best-performing impact absorption formulations. These will be applied to composite structures and tested in real prototypes, such as knee and elbow pads. Once the optimized performance is validated, a survey will be conducted with soldiers in real-world conditions to assess the comfort and acceptance of these functionalized prototypes.

## Figures and Tables

**Table 1:** Produced samples.

Structure	Impregnated Matrice	Matrice Percentage (wt%)	Resin + STF Ratio (wt%)	Sample Name
Ref. sponge	-	-	-	Ref_sponge
Auxetic	-	-	-	Ref_aux
	STF	200	(100:0)	Aux_200%STF
		400	(100:0)	Aux_400%STF
	Biresin	200	(100:0)	Aux_200%Bir
		400	(100:0)	Aux_400%Bir
	Biresin + STF	200	(75:25)	Aux_200%(Bir + STF)
		400	(75:25)	Aux_400%(Bir + STF)

**Table 2:** Significant bands verified on FTIR spectra.

	Wavenumber cm^−1^	Assignment	Type of Vibration
A	~3400	O-H (alcohol)	stretch
B	~2920	C-H (asym, stretch)	asym, stretch
C	~1711	C=O aldehyde	stretch
D	~1626	C=C alkene	stretch
E	~1528	Amide II (amide N-H bend)	bend
F	~1385	C-H sym	sym, stretch
G	~1230	C-O aliphatic ether	stretch
H	~1093	C-O aliphatic ether	stretch

## Data Availability

The original contributions presented in this study are included in the article. Further inquiries can be directed to the corresponding author.
